# PAMAM Dendrimers Cross the Blood–Brain Barrier When Administered through the Carotid Artery in C57BL/6J Mice

**DOI:** 10.3390/ijms18030628

**Published:** 2017-03-14

**Authors:** Bhairavi Srinageshwar, Sarah Peruzzaro, Melissa Andrews, Kayla Johnson, Allison Hietpas, Brittany Clark, Crystal McGuire, Eric Petersen, Jordyn Kippe, Andrew Stewart, Olivia Lossia, Abeer Al-Gharaibeh, Aaron Antcliff, Rebecca Culver, Douglas Swanson, Gary Dunbar, Ajit Sharma, Julien Rossignol

**Affiliations:** 1 Field Neurosciences Institute laboratory for Restorative Neurology at Central Michigan University, Mt. Pleasant, MI 48859, USA; srina1b@cmich.edu (B.S.); peruz1st@cmich.edu (S.P.); andre2mm@cmich.edu (M.A.); mcgui4c@cmich.edu (C.M.); kippe1jm@cmich.edu (J.K.); stewa2an@cmich.edu (A.S.); lossi1ov@cmich.edu (O.L.); algha2a@cmich.edu (A.A.-G.); antcl1ak@cmich.edu (A.A.); culve1rl@cmich.edu (R.C.); dunba1g@cmich.edu (G.D.); 2 Program in Neuroscience, Central Michigan University, Mt. Pleasant, MI 48859, USA; peter1ed@cmich.edu; 3 Department of Chemistry & Biochemistry Central Michigan University, Mount Pleasant, MI 48859, USA; johns52k@cmich.edu (K.J.); hietp1ar@cmich.edu (A.H.); clark6bm@cmich.edu (B.C.); swans1d@cmich.edu (D.S.); sharm1a@cmich.edu (A.S.); 4 Field Neurosciences Institute, St. Mary’s of Michigan, Saginaw, MI 48604, USA; 5 College of Medicine, Central Michigan University, Mt. Pleasant, MI 48859, USA; 6 Department of Psychology, Central Michigan University, Mt. Pleasant, MI 48859, USA

**Keywords:** PAMAM dendrimer nanoparticle, blood–brain barrier, non-invasive delivery, bio-distribution and uptake, neurodegenerative diseases

## Abstract

Drug delivery into the central nervous system (CNS) is challenging due to the blood–brain barrier (BBB) and drug delivery into the brain overcoming the BBB can be achieved using nanoparticles such as dendrimers. The conventional cationic dendrimers used are highly toxic. Therefore, the present study investigates the role of novel mixed surface dendrimers, which have potentially less toxicity and can cross the BBB when administered through the carotid artery in mice. In vitro experiments investigated the uptake of amine dendrimers (G1-NH_2_ and G4-NH_2_) and novel dendrimers (G1-90/10 and G4-90/10) by primary cortical cultures. In vivo experiments involved transplantation of G4-90/10 into mice through (1) invasive intracranial injections into the striatum; and (2) less invasive carotid injections. The animals were sacrificed 24-h and 1-week post-transplantations and their brains were analyzed. In vivo experiments proved that the G4-90/10 can cross the BBB when injected through the carotid artery and localize within neurons and glial cells. The dendrimers were found to migrate through the corpus callosum 1-week post intracranial injection. Immunohistochemistry showed that the migrating cells are the dendrimer-infected glial cells. Overall, our results suggest that poly-amidoamine (PAMAM) dendrimers may be used as a minimally invasive means to deliver biomolecules for treating neurological diseases or disorders

## 1. Introduction

Dendrimers are well-defined 3-dimensional highly branched polymeric nanoparticles that are being widely used in various biomedical applications. They are typically composed of three major components: (1) the core; (2) the interior layers, or the generations (G), made of repeated units; and (3) the shell, or the exterior, which may terminate in a variety of functional groups (such as the cationic amines, anionic carboxyls, or neutral hydroxyls) under the typical cellular pH of 7. Though there are a wide variety of dendrimers available, a popular and commercially available class of dendrimers is poly-amidoamine (PAMAM) dendrimers [1,2] which have an amide backbone similar to proteins with various cores and surface groups. Major advantages of PAMAM dendrimers with specific surfaces are (1) biocompatibility; (2) non-immunogenicity; and (3) water solubility, which is highly suitable for drug or gene delivery [3]. Certain PAMAM dendrimers also have anti-inflammatory properties and can confer therapeutic effects for a long period of time [4]. Due to their small size and high water solubility, PAMAM dendrimers are readily eliminated from the body via renal excretion [5]. As a vehicle, PAMAM dendrimers can carry a specific amount of DNA/gene or drug that can be targeted to cells, both in vitro and in vivo. The amount of cargo the dendrimers can carry depends on generation (G) of the dendrimers, G1 to G3 being planar in structure compared to higher order dendrimers [6].

There are previous studies that described the ability of the dendrimers to enter the cells. Albertazzi and colleagues (2013) showed in vitro permeability of ethylenediamine (EDA) core G4 amine and G4-C12 PAMAM dendrimers into primary neurons. These dendrimers can be used as a vehicle to transfect cells with small-sized DNA/RNA, without the use of any conventional transfecting agents [7]. Generally, the electrostatic interaction between the cationic dendrimer and the anionic cell membrane facilitates the uptake of dendrimers by the cells [8]. However, a major issue with drug delivery into the central nervous system (CNS) is the presence of blood–brain barrier (BBB), which is a complex multicellular structure that is selectively permeable to molecules and separates the CNS from the circulatory system [9]. The BBB consists of tight junctions made of mono-layered endothelial cells that prevent leakage of plasma into the CNS [10]. Molecules or drugs that are less than 1 nm in size are capable of passing through the BBB [11]. Lipids and lipid-based carrier molecules of larger sizes can also cross the BBB by diffusing through the tight junctions, but the rate of drug delivery by this means is highly reduced. It is difficult for most of the potential drugs or the biomolecules to enter a specific site in the brain [10] unless directly injected into the brain. The disadvantages of the current pharmacological therapies include drug insolubility, reduced bioavailability, and low specificity [5].

In order to deliver drugs to targeted areas within the brain, intracranial injection has been the predominant method used. However, one of the major drawbacks of intracranial injections is that the procedure is highly invasive and the delivery is often localized to the administration site and the drug administration cannot be given if multiple treatments are needed [12]. This method may be advantageous for targeted areas, such as tumors, but would not deliver to a wide-range of neural tissue, as would be needed to treat neurodegenerative diseases, like Alzheimer’s disease (AD) or Huntington’s disease (HD). Direct invasive injections into the brain also lead to increased risk, as well as more variability in the amount of drug being delivered over time [13]. Hence, with any potential treatment, route of administration and the surface chemistry of the drug plays a critical role in their efficacy and clinical utility [14].

To overcome the pitfalls associated with the invasive intracranial injection, carotid injections offer an alternative method of delivering drugs into the brain without opening the skull and inserting needles into the brain tissue. Previous studies have shown that amine dendrimers can cross the artificial BBB, in vitro, and enter endothelial cells and primary neural cells. Since injections into the carotid artery go directly to the brain, the first pass metabolism is avoided and absorption into peripheral tissues are reduced [15]. Drugs infused into the internal carotid artery (ICA) attain high initial arterial concentrations, even at low total doses of drugs, because of low volume of blood flow. Drugs delivered through this route of administration can attain not only high drug concentrations in the brain, but do so instantaneously [16]. Carotid injections of different drugs have also been used for treating ischemic strokes, intractable cerebral vasospasm, and intracranial malignancies [17].

**Figure 1:**
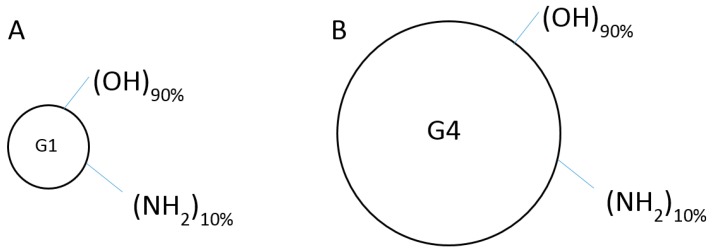
(**A**) G1-90/10 dendrimer having 90% of surface covered with OH (neutral) groups and 10% of surface covered with NH_2_ (cationic) groups; (**B**) G4-90/10 dendrimer having 90% of surface covered with OH (neutral) groups and 10% of surface covered with NH_2_ (cationic) groups.

Vidal and Guzman (2015), and De Stefano and colleagues (2012) warned about the potential toxic in vivo effect of NH_2_ surface dendrimers that have abundant cationic amine groups, such as the 64 amines found on a G4 dendrimer [5,18]. They were toxic in a dosage-dependent manner, causing holes on the lipid membranes of the cells, ultimately leading to cell death [13,18,19,20,21]. Fortunately, PAMAM dendrimers can easily be designed to reduce or eliminate potential toxic effects by reducing the surface amine groups and replacing them with a more biocompatible group, such as the hydroxyl (–OH) moiety. To reduce the possibility of this dendrimer-induced toxicity, we synthesized G1 (~1 nm; molecular weight (MW) of ~1458 Da) and G4 (~4 nm; MW of ~14,300 Da) dendrimers, with diaminobutane (DAB) core with only 10% of the surface covered with cationic amine groups. The remaining 90% of their surface consisted of neutral hydroxyl groups. A representation of these mixed surface neutral/cationic (90%-OH/10%-NH_2_ surface; G1-90/10; G4-90/10) dendrimers is shown in Figure 1.

The primary rationale of the study is to validate, as a proof of concept, if our novel mixed surface dendrimers are able to cross the BBB, which in future can be used to deliver drugs/biomolecules by less invasive methods. Our lab has a strong focus on Huntington’s disease (HD), which is caused by degeneration of medium spiny neurons (MSNs) in the striatal region of the brain. To be consistent, we examined striatal delivery of dendrimers and compared it to the systemic delivery using the carotid. In the present study, both G1 and G4 amine surface dendrimers as well as mixed surface dendrimers, were used for in vitro uptake experiments by primary cortical neurons and glial cells. However, in order to avoid toxicity of purely amine surface dendrimers, as described in various studies [18,19,20,21], only the G4-mixed surface dendrimers were used for in vivo experiments that assessed the ability of the dendrimers to cross the BBB of C57BL/6J mice when administered through the carotid artery. Moreover, the G1 dendrimer (both the 100% amine surface and G1 90/10) has a 2-dimensional planar structure [6] which makes them incapable of carrying any cargo and were used only in in vitro experiments. G4 dendrimers are suitable for accommodating cargo due to their three-dimensional structure and were used in vitro and in vivo.

## 2. Results

### 2.1. Dendrimer Characterization

**Figure 2:**
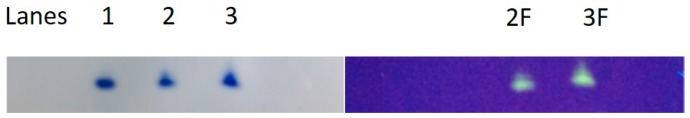
Acidic gel electrophoresis of dendrimers. **Lane 1** represents unlabeled G4-90/10; **Lane 2** represents G490/10 reacted with 50% mol fluorescein isothiocyanate (FITC) added compared to the mol of amine groups in the dendrimer (**2F**); **Lane 3** represents G4-90/10 reacted with 100% mol FITC added compared to the mol of amine groups in the dendrimer (**3F**). Since there was not much difference in the intensity between the two, we decided to use the dendrimer prepared shown in lane 2 to avoid decreasing the conjugate’s aqueous solubility by adding more FITC.

**Figure 3:**
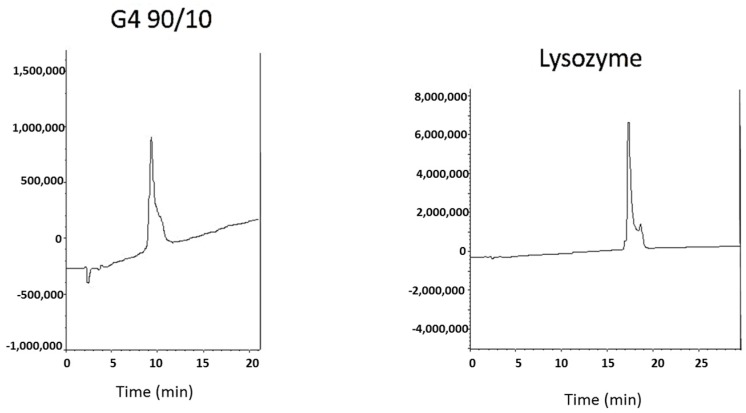
Reversed phase high performance liquid chromatography (RP-HPLC) of dendrimers: The retention time for G4-90/10 surface is 10.1 min and the retention time for Hen egg white lysozyme is 18 min. Comparable peak size between G4-90/10 dendrimers and lysozyme shows purity of the dendrimers.

**Figure 4:**
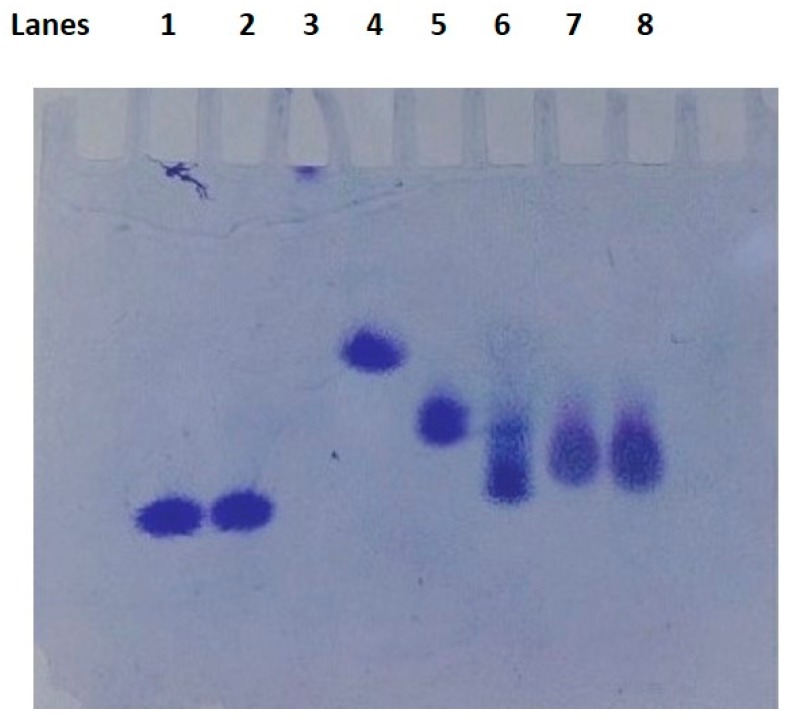
Isoelectric focusing (IEF) of G4-90/10 dendrimer. The top half of the gel is acidic and the bottom half is alkaline. Lanes 1 and 2 are lysozyme (pI 11.0); lane 3 is amyloglucosidase (pI 3.5; protein barely entered the gel); lane 4 is myoglobin (pI 6.8), lane 5 is ribonuclease A (pI 9.5), lane 6 is cytochrome c (pI 10.7); and lanes 7 and 8 are G4-90/10 dendrimers.

Dendrimers were characterized using acidic native polyacrylamide gel electrophoresis (PAGE), isoelectric focusing (IEF), and reverse phase-high performance liquid chromatography (RP-HPLC). Typical results are shown in Figure 2, Figure 3 and Figure 4. Acidic native PAGE showed one band for the G4-90/10 dendrimer and its fluorescent conjugate when stained with Coomassie Brilliant Blue (Figure 2; lanes 1–3). Attachment of fluorescein isothiocyanate (FITC) clearly showed the fluorescent dendrimer under ultraviolet light (Figure 2; lanes 2F,3F). RP-HPLC (Figure 3) showed relatively pure G4-90/10 dendrimer (retention time 10.1 min) when compared to a cationic protein of comparable size (lysozyme; retention time 18 min). IEF showed that the G4-90/10 (Figure 4; lanes 7 and 8) migrated between cytochrome c (with isoelectric point (pI) 10.7) and ribonuclease A (pI 9.5). The pI of the G4-90/10 and G1-90/10 dendrimers were calculated to be 10 and 11, respectively, from a calibration plot of migration distance versus pI of standard proteins (data not shown). The mixed surface dendrimers thus behave as basic proteins with respect to their isoelectric points (pI).

### 2.2. In Vitro Primary Cortical Culture Infection with Dendrimers

#### 2.2.1. Cationic Dendrimers

**Figure 5:**
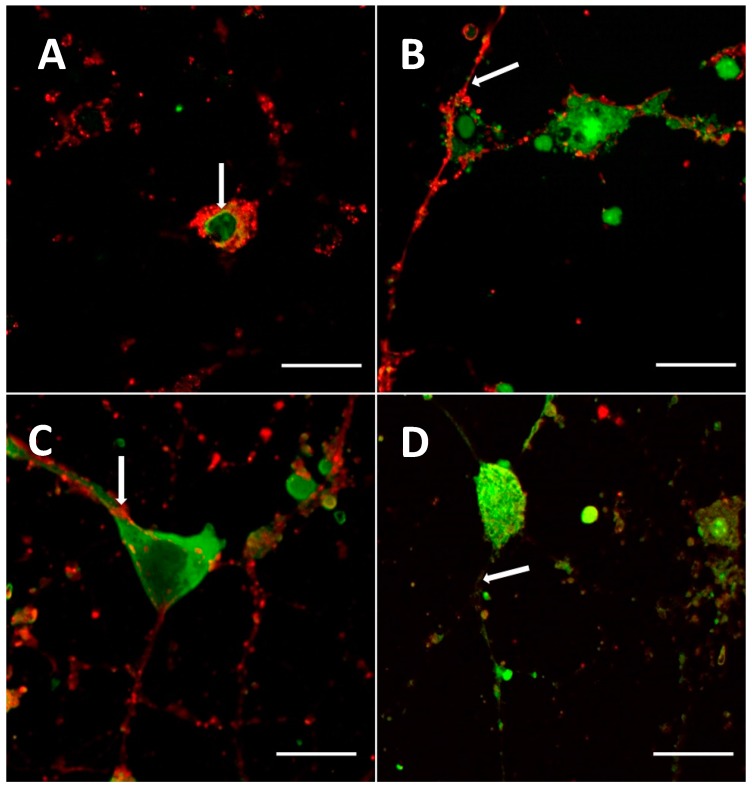
Confocal images showing cellular uptake of (**A**) G1-NH_2_; (**B**) G1-90/10; (**C**) G4-NH_2_; and (**D**) G4-90/10 dendrimers by the primary cortical culture (neurons and glial cells). Co-localization between the cellular stain (red) and the FITC labeled dendrimers (green) were observed. The processes of the neurons were also able to uptake the dendrimer (arrow; **A**–**D**). All images were taken at 60× (2.5× zoomed in) magnification using Olympus BX50 Upright Microscope (Olympus, Shinjuku, Tokyo, Japan). Scale bar: 20 μm.

Uptake of G1-NH_2_ and G4-NH_2_ dendrimers by primary cortical culture (neurons and glial cells) was observed at a concentration of 0.5 mg/mL when incubated for 30 min. Co-localization between the cellular stain PKH26 Red Fluorescent Cell Linker (Sigma Aldrich, St. Louis, MO, USA), and the G1-NH_2_ and G4-NH_2_ dendrimers were observed, confirming that the dendrimers were able to infect the cells and migrate into the nucleus, cytoplasm, and the processes of the cells (Figure 5A,C).

#### 2.2.2. Mixed Surface Dendrimers

Uptake of G1-90/10 and G4-90/10 dendrimers by primary cortical culture (neurons and glial cells) was observed at the concentration of 4 mg/mL when incubated for 30 min. Co-localization between the cellular stain and the G1-90/10 and G4-90/10 dendrimers was observed, confirming that the dendrimers were able to infect the cells and migrate specifically into the nucleus, cytoplasm, and the processes of the cells, as shown in Figure 5B,D.

### 2.3. Localization of Dendrimers In Vivo

#### 2.3.1. Carotid Injection of Dendrimers

**Figure 6:**
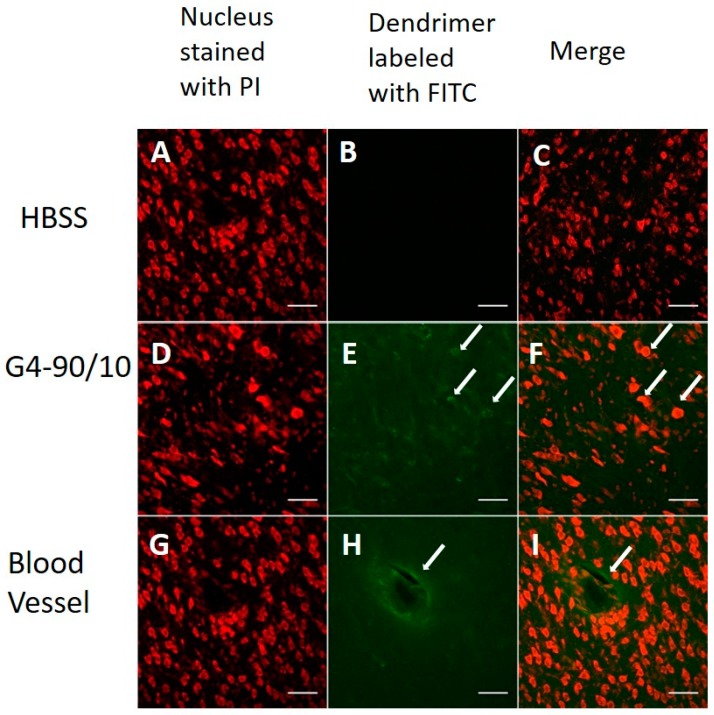
Confocal images of dendrimer in the brain when injected through the carotid artery. Neurons and glial cells labeled with propidium iodide (PI) stain (red—**A**,**D**,**G**), Hank’s balanced salt solution (HBSS) control and merge (**B**,**C**), G4-90/10 infecting cells (arrow; **E**) and merge (arrow; **F**), G4-90/10 surrounding the blood vessel (arrow; **H**) and merge (arrow; **I**). All images were taken at 40× magnification using Olympus BX50 Upright Microscope (Olympus, Shinjuku, Tokyo, Japan). Scale bar: 100 µm.

The G4-90/10 dendrimers were able to cross the BBB, as shown in Figure 6, and were able to infect the cells. Co-localization between the FITC dendrimer and cellular stain was observed (E and F). The dendrimers were found around blood vessels and diffusing out to the surrounding brain tissue (H and I), confirming their ability to cross the BBB. In addition, there was no detectable amount of dendrimer in the liver, lungs, and spleen tissue sections (data not shown). Hence, we conclude that injection via the carotid route is an appropriate delivery of dendrimers to the brain.

#### 2.3.2. Intracranial Injection of Dendrimers

**Figure 7:**
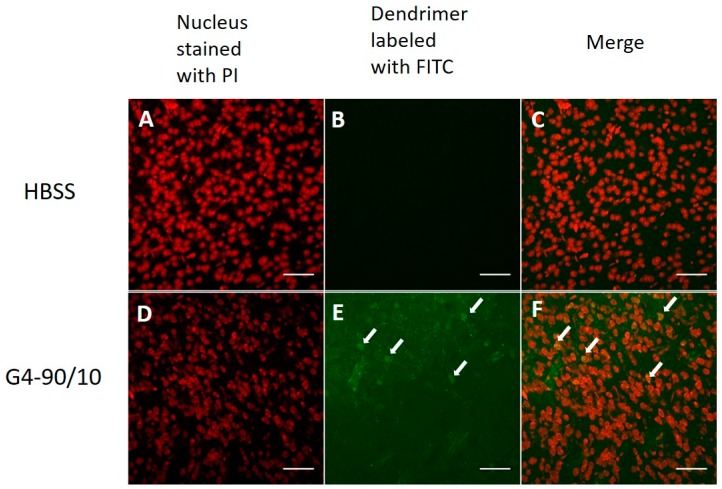
Confocal images of dendrimer in the brain when injected intracranially. Neurons and glial cells labeled with propidium iodide stain (red—**A**,**D**), HBSS control and merge (**B**,**C**), G4-90/10 infecting cells (arrow; **E**) and merge (arrow; **F**). All images were taken at 40× magnification using Olympus BX50 Upright Microscope (Olympus, Shinjuku, Tokyo, Japan). Scale bar: 100 μm.

**Figure 8:**
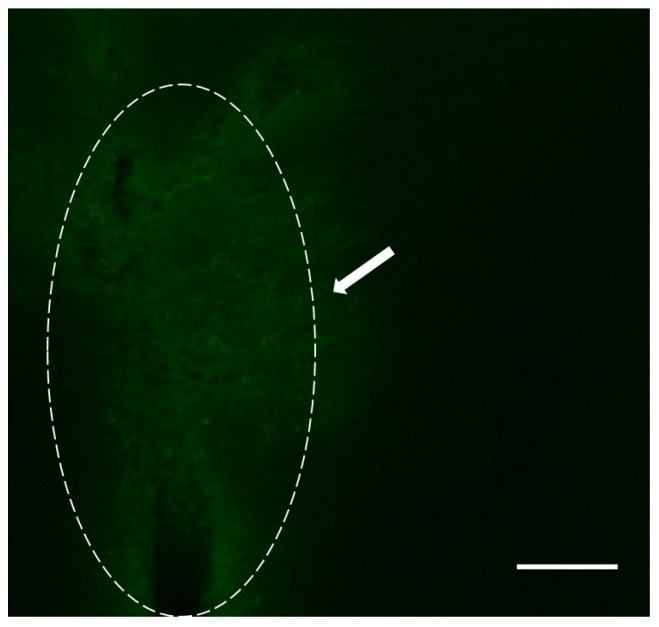
Confocal image of G4-90/10 dendrimer surrounding the needle tract (arrow pointing to the region). Image was taken at 40× magnification using Olympus BX50 Upright Microscope (Olympus, Shinjuku, Tokyo, Japan). Scale bar: 100 µm.

The G4-90/10 dendrimers were found in the striatal region of the brain post 24-h, as shown in Figure 7. Co-localization between the FITC dendrimer and cellular stain was observed (E and F). In the case of intracranial injection into the striatum, the FITC dendrimer was found surrounding the needle tract, confirming the presence of dendrimer at the injection site (Figure 8).

### 2.4. Histology

#### Dendrimer Migration 1-Week Post-Transplantation and Co-Localization with Neurons and Glial Cells

**Figure 9:**
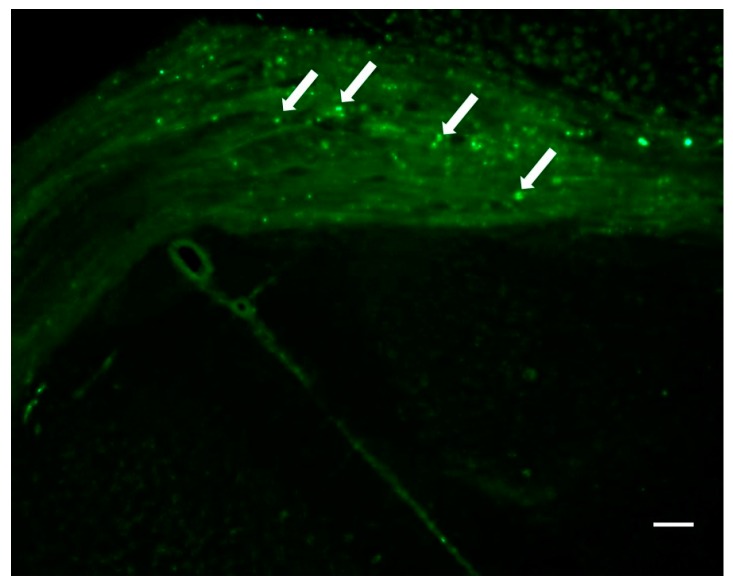
Image of G4-90/10 dendrimer migrating across the hemispheres through the corpus callosum (arrow) 1-week post-intracranial transplantation into the striatum. Image was taken at 10× magnification using Zeiss Observer inverted Microscope (Zeiss, Oberkochen, Germany) Scale bar: 100 μm.

**Figure 10:**
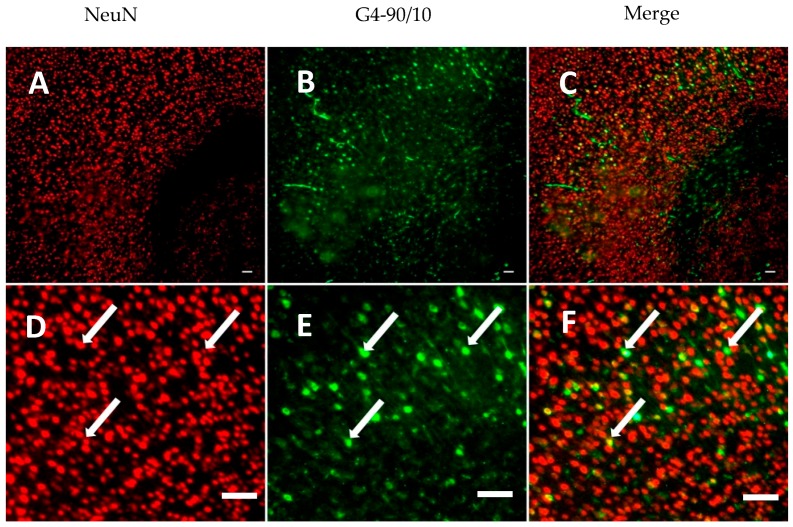
(**A**) Nucleus stained with NeuN antibody (red); (**B**) uptake of G4-90/10 dendrimers by the neurons in the cortex 1-week post intracranial transplantation; (**C**) Co-localization between NeuN and dendrimers showing uptake of dendrimers by the neurons; (**D**–**F**) show the zoomed images of (**A**–**C**), respectively. Arrows in **D** shows neurons expressing NeuN; arrows in **E** shows uptake of G4-90/10 dendrimers by the neurons and arrows in **F** shows co-localization between the dendrimers and NeuN staining that confirms the uptake of dendrimers by neurons. Images (**top panel**) were taken at 20× magnification using Zeiss Observer inverted Microscope (Zeiss, Oberkochen, Germany). Scale bar: 100 μm.

**Figure 11:**
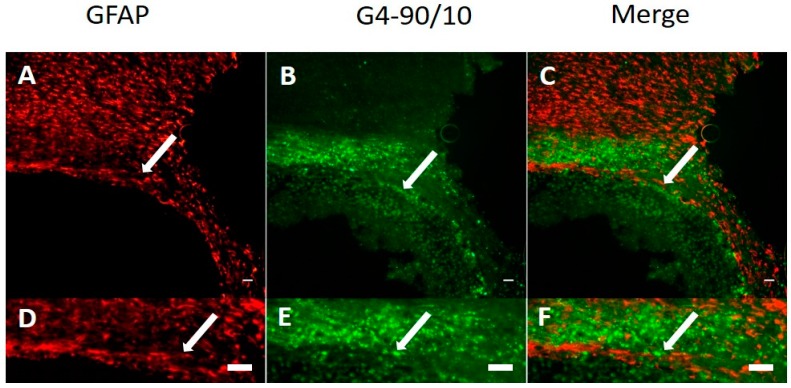
(**A**) Glial cells stained with Glial fibrillary acidic protein (GFAP) antibody (red; see arrow); (**B**) uptake of G4-90/10 dendrimers by the glial cells especially in the corpus callosum 1-week post-intracranial transplantation (arrow); (**C**) Co-localization between GFAP and dendrimers showing uptake of dendrimers by the glial cells (arrow); (**D**–**F**) show the zoomed images of (**A**–**C**), respectively, at the corpus callosum region. Arrow in **D** shows glial cells expressing GFAP; arrow in **E** shows uptake of G4-90/10 dendrimers by the glial cells and arrow in **F** shows co-localization between the dendrimers and GFAP staining that confirms the uptake of dendrimers by glial cells. This proves that the migrating cells are the glial cells that have taken up the dendrimers. Images (**top panel**) were taken at 20× magnification using Zeiss Observer inverted Microscope (Zeiss, Oberkochen, Germany). Scale bar: 100 μm.

The G4-90/10 dendrimers were found to migrate from one hemisphere to another through the corpus callosum, 1-week post-intracranial injection into the striatum. The dendrimers were able to infect cells in the brain including the cortical regions, as shown in Figure 9. Neuronal nuclei (NeuN) and Glial fibrillary acidic protein (GFAP) staining showed that the dendrimers co-localized with neuronal nuclei and glial cells, indicating that both neurons and glial cells can uptake the dendrimers (Figure 10 and Figure 11). The finding that the dendrimers which crossed the hemispheres through the corpus callosum were co-localized with the glial cells suggests that the migrating cells are the glial cells that had taken up the dendrimers (Figure 11).

## 3. Discussion

Our in vitro study showed that both NH_2_ and mixed surface dendrimers of different sizes can infect the cultured neuronal cells within a short period of time. However, longer durations of dendrimer infection in vitro and in vivo should be studied in the future. The dendrimers were found in the nucleus and the cytoplasm of the cells in vitro, as shown in Figure 5. As these dendrimers (especially G4) are able to reach the nucleus, they can be used for carrying cargo, which can be delivered into the cells. Since the 100% NH_2_ surface dendrimers have been reported to be toxic, our mixed surface G4 dendrimer (with only 10% NH_2_ surface) offers a promising alternative for delivering cargo such as drugs or therapeutic molecules in vivo. Generally, uptake of the dendrimer by the cells is dependent on the electrostatic interaction between the positive charge on the surface of the dendrimer and the negative charge on the surface of the cell membrane [9]. Since the 100% NH_2_ surface dendrimer has more positive charges, compared to the 90% OH and 10% NH_2_ surface dendrimer, the former enters the cell quicker and at a lower concentration, compared to the mixed surface dendrimers in vitro.

An important in vivo finding of this study is that our mixed surface dendrimer can cross the blood–brain barrier when injected through the carotid artery and can target and “infect” the neurons and glial cells in the brain. The migration of dendrimers at 1-week post-intracranial transplantation shows that the brain was able to retain the dendrimers and that they were able to infect the neurons and glial cells in the cortex and other regions of the brain. No tissue necrosis was found. The glial cells that took up the dendrimers were migrating across the corpus callosum. However, further investigations addressing the effect of dendrimer infection at different time points and analyzing the extent of migration is crucial. A major application of this finding is that these dendrimers can be used as a vehicle to carry drugs that cannot cross the blood–brain barrier.

Some of the previous studies have used dendrimers to deliver drugs and/or biomolecules which has been successfully translated into clinical settings. According to a 2014 report, there are two dendrimer based drugs in clinics used as a treatment for bacterial vaginosis and metastatic cancer [22]. The dendrimers such as paramagnetic iron oxide particles (magneto-dendrimers) and gadolinium-chelates are used for imaging in clinics. Dendrimers have also been linked to some monoclonal antibodies that can be used for targeted drug delivery and imaging purposes. The dendrimers are capable of carrying cargo such as DNA and RNA molecules [23]. In 2001, dendrimer based anti-sense oligo DNA was delivered to target and image intraperitoneal tumors [24].

Although previous studies have used 100% NH_2_ surface dendrimers and other dendrimers for drug delivery, there was evidence of toxicity with those dendrimers [5,18]. Our mixed surface dendrimers have been synthesized de novo from a DAB core to form a surface with 10% NH_2_ and 90% OH groups. The ten-fold reduction in surface amines in these dendrimers is expected to drastically reduce their toxicities, but allow them to be easily labeled by fluorescent dyes and readily bind to cell surfaces, as shown by cellular uptake studies. IEF reveals that, with respect to pI values, the mixed surface dendrimers behave as basic proteins, such as cytochrome c and lysozyme, which are known to bind cell membranes. Cells that were infected with the dendrimers were still viable, suggesting the non-toxic nature of these nanomolecules. Lesniak and colleagues (2013) used G4-100% OH dendrimers for their study. However, the dendrimers were modified to have three NH_2_ groups on their surface, which enabled the dendrimers to be labeled with a fluorescent molecule. The dendrimers were injected through the tail-vein and most of the dendrimers in that investigation accumulated in peripheral organs, rather than reaching the brain [25]. Therefore, injection of our dendrimers through the carotid artery proved to be a less invasive and a more successful strategy to deliver the dendrimers, since most of the injected dendrimers reached the brain, rather than being concentrated in the peripheral circulation. In the future, using intra-carotid injection for delivery of dendrimers may be one of the most potent applications for the treatment of brain disorders [16]. In our novel dendrimer, 10% NH_2_ group represent five to six NH_2_ out of which the FITC dye can be attached to usually two or three NH_2_. The rest of the amines (two or three) are free, which will increase the potential for the dendrimer to enter the cell membranes. Also, the fluorescent dyes are hydrophobic in nature, which facilitates the intake of dendrimer by the cells to some extent. To the best of our knowledge, there are no reports of using G4-100% OH in an unmodified form. Though it may be possible for a G4-100% OH dendrimer to enter the cells, we were not able to track them down and confirm the cell entry.

In conclusion, we confirmed that our mixed surface dendrimers can cross the BBB when injected by systemic route, and can infect the brain cells which confer to them a potential to deliver drugs inside the brain.

## 4. Materials and Methods

### 4.1. Dendrimer Synthesis and Characterization

#### 4.1.1. PAMAM Dendrimer Synthesis G1 and G4, DAB Core, 100% NH_2_ Surface

PAMAM dendrimers were synthesized as previously reported [26]. The general synthesis involves a reiteration of the alkylation of an amine with methyl acrylate, followed by the amidation of the methyl ester with high excess ethylenediamine. This process is repeated throughout the preparation, giving a new generation per each reiteration. Thus, the first reiteration from a core amine yields generation 0 (G0) and the next reiteration will yield generation 1 (G1), and so on. All dendrimers used in this study contained a diaminobutane (DAB) core.

#### 4.1.2. Preparation of PAMAM Mixed Surface Dendrimer G = 1, DAB Core, 90% OH, 10% NH_2_ (G1-90/10)

Ethanolamine (36 g, 0.59 mol; 90 equivalents per ester) and ethylenediamine (3.9 g, 0.065 mol; total 10 equivalents per ester) and 7 mL methanol was added to a 50 mL round-bottom flask containing a stir bar. A PAMAM dendrimer having a DAB core, G = 0.5, (MW = 1233; 1.0 g; 0.81 mmol dendrimer; 6.5 mmol ester) was added to this mixture (cooled to 8 °C) in 2 mL methanol, dropwise, over 5 min. This mixture was stirred for 10–15 min and placed in a refrigerator at 8 °C for 10 days. An aliquot of this mixture was analyzed by infrared spectroscopy to validate the complete disappearance of the ester carbonyl group at 1735/cm. The mixture was allowed to warm to room temperature and stirred overnight. Volatiles in the mixture were removed using a bulb-to-bulb distillation with a high vacuum and 60–85 °C. The resulting residue was dissolved in methanol and purified using a Sephadex LH-20 column eluted with methanol. Fractions of 20 mL were taken and those containing a dendrimer product (determined by spotting each fraction on a TLC (thin layer chromatography) plate and developing the plate in an iodine chamber) were collected, and more volatiles were removed using a rotary evaporator, followed by high vacuum at 35 °C. This dendrimer fraction was further stripped of volatiles on a rotary evaporator, followed by high vacuum at 40 °C for 30 min to give 1.1 g (92% yield, MW = 1458) of the desired product.

#### 4.1.3. Preparation of PAMAM Mixed Surface Dendrimer G = 4, DAB Core, 90% OH, 10% NH_2_ (G4-90/10)

Ethanolamine (29.3 g, 0.48 mol; 100 equivalents per ester) and ethylenediamine (3.2 g; 0.05 mol; total 100 equivalents per ester) and 7 mL methanol were added to a 50 mL round-bottom flask containing a stir bar. A PAMAM dendrimer having a DAB core, G = 2.5 (MW = 6039; 1.0 g; 0.166 mmol; 5.3 mmol ester) was added to this mixture (cooled at 8 °C) in 2 mL methanol, dropwise, over 5 min. This mixture was stirred for about 10–15 min and placed in a refrigerator at 8 °C for 10 days. An aliquot of the mixture was analyzed by infrared spectroscopy to confirm the complete disappearance of the ester carbonyl group at 1735/cm. This mixture was allowed to warm to room temperature and stirred overnight. The mixture was diluted to 600 mL with methanol and purified using tangential flow ultrafiltration apparatus, containing 3 KDa cutoff regenerated cellulose membranes. After the first recirculation (650 mL), the retentate volume was reduced to ~400 mL. After the second recirculation (400 mL), the retentate volume was reduced to 200 mL. This process was repeated until the retentate volume was ~30 mL. A total of 10 re-circulations were done or until a TLC of the mixture (silica gel, 5% NH_4_OH in MeOH *v*/*v*) indicated the complete disappearance of ethanolamine. This resulting mixture was stripped of volatiles on a rotary evaporator followed by high vacuum at 40 °C for 30 min to give 11 g (95% yield, MW = 14,300) of the desired product. All the NH_2_ dendrimers were re-suspended in phosphate buffered saline (PBS; pH 7.4) while the mixed surface dendrimers were re-suspended in Hank’s balanced salt solution (HBSS; Gibco Waltham, MA, USA).

#### 4.1.4. Preparation of Dendrimer-FITC Conjugates

Since all of the dendrimers used in this study had surface amines, they were readily labeled with fluorescein isothiocyanate (FITC). The dendrimer was reacted with FITC (dissolved in dimethylformamide; DMF) in bicarbonate buffer (0.5 M, pH 9.7). In the case of dendrimers, G1 and G4 with 100% NH_2_ surface, the amount of FITC added was 5% compared to the amount of amine groups present in the dendrimer. In the case of mixed surface dendrimers, G1-90/10 and G4-90/10, the amount of FITC added was 50% compared to the amount of amine groups in the dendrimer. The reaction was stirred for 1 h in the dark. In the case of G1 dendrimers, the conjugates were purified by dialysis with 100–500 Da molecular weight cut-off (MWCO) Float-A-Lyzer G2 dialysis device (Spectrum Labs, Houston, TX, USA). G4 dendrimer-FITC conjugates were purified by using 3 KDa MWCO Nanosep Omega centrifugal devices (Pall Corporation, Ann Arbor, MI, USA).

#### 4.1.5. Characterization of Dendrimers

Dendrimers and their conjugates were analyzed by acidic gel electrophoresis, as previously described by Sharma and colleagues [27], and isoelectric focusing by Upadhaya and colleagues [28], and reversed phase HPLC. IEF was performed using high resolution pH 3–10 ampholyte solution (Fluka) with 10 mM phosphoric acid and 20 mM NaOH. HPLC was performed on a Hitachi system with an autosampler (L-7200), pump (L-7100), UV-visible detector (L-7420), and an interface (D-7000) with a 20-µL loop. The C18 column used was a Varian Microsorb-MV (250 mm × 4.6 mm, 300 Å) attached to a MetaGuard 4.6 mm Microsorb 300 Å 5 µ C18 guard column (Agilent Technologies, Santa Clara, CA, USA). Mobile phase A was 0.1 wt % aqueous TFA (Trifluoroacetic acid), and mobile phase B was 0.085 wt % TFA in acetonitrile. A linear gradient elution was employed, starting with 5% B and ending 90% B in 30 min, at a flow rate of 1 mL/min. Samples were prepared in HPLC grade water and filtered through a 0.45 µm syringe filter.

### 4.2. In Vitro

#### 4.2.1. Extraction of Primary Cortical Neurons from Mouse (E18)

Pregnant C57BL/6J female mice (Jackson Laboratory, Bar Harbor, ME, USA) at gestation 18 days were sacrificed using CO_2_. A laparotomy was performed to expose the uterine horn which was surgically opened. The embryos were removed and decapitated one at a time using sharp surgical scissors and washed with ice-cold HBSS (Gibco, Waltham, MA, USA) having 1% penicillin streptomycin (P/S, Gibco). From each of the decapitated embryos, the brain was extracted and the cortex was separated out under the dissection microscope. The cortex was collected in a 35-mm petri dish and transferred to a 15-mL tube containing ice-cold HBSS with 1% P/S. The tissue was allowed to settle down and the supernatant was pipetted off. The tissue was re-suspended in 2 mL of Neurobasal media (Gibco, Waltham, MA, USA) containing B27 with vitamin A, Glutamax, P/S, MEM-(minimum essential medium) with non-essential amine acids (NEAA) (Gibco, Waltham, MA, USA; known as primary cortical neuron (PCN) media) and pipetted to achieve single cell suspension. The un-dissociated pieces of tissue were allowed to settle and the supernatant was transferred to a new 15 mL tube and centrifuged at 150× *g* for 5 min. The supernatant was carefully aspirated and the cell pellet was re-suspended in 2 mL PCN media and counted using hemocytometer. 24-h prior to plating the cells, the coverslips were coated with 0.2 mg/mL Poly-l-Lysine (Sigma Aldrich, St. Louis, MO, USA). The cell viability was assessed using trypan blue (Thermo Fisher Scientific, Waltham, MA, USA) staining method. The cells were plated at a density of 2 × 10^5^ viable cells/mL. Partial medium change was done once a week.

#### 4.2.2. Dendrimer Permeability Assay with Primary Cortical Culture

The primary cortical culture was allowed to grow for 14 days after which they were infected with G1-NH_2_, G4-NH_2_, G1-90/10, and G4-90/10 dendrimers. These dendrimers were used at various concentrations incubated at 37 °C at three different time points to optimize the cellular uptake (data not shown). The final concentration of NH_2_ surface dendrimer was optimized to be 0.5 mg/mL and mixed surface dendrimer was optimized to be 4 mg/mL, which were taken up by the cultured cortical culture cells (neurons and glial cells) in 30 min. The dendrimers were added to the cells after labeling the cells with PKH26 Red Fluorescent Cell Linker (Sigma Aldrich, St. Louis, MO, USA), according to the manufacturer’s protocol. The labeled culture cells were fixed with 4% paraformaldehyde (PFA), mounted, and viewed under Zeiss Observer inverted microscope, and confocal images were captured using Olympus BX50 Upright Microscope.

### 4.3. In Vivo

#### 4.3.1. Animals

A total of twenty-one C57BL/6J mice aged between 6 and 15 weeks (Jackson Laboratory, Bar Harbor, ME, USA) were used in this study. All the procedures associated with animals followed the guidelines of the Institutional Animal Care and Use Committee (IACUC) of Central Michigan University (28 August 2015, and was registered under the CMU IACUC protocol #15-29). All the C57BL/6J mice were housed in clear polycarbonate cages, with three or four mice per cage, and at 22 °C and under 12-h light/12-h dark cycle. The animals were given access to food and water ad libitum. Both male and female animals were included in this study.

#### 4.3.2. Groups

The animals received 4 μL of 10 mg/mL G4-90/10 dendrimer or 4 μL of vehicle, either by intracranial or carotid injection. The animals were randomly divided into four major groups: (1) animals receiving G4-90/10 dendrimers by intracranial injection (*n* = 3); (2) animals receiving G4-90/10 dendrimers by carotid injection (*n* = 4); (3) control animals receiving vehicle by intracranial injection (*n* = 3); and (4) control animals receiving vehicle by carotid injection (*n* = 2). The above groups of animals were sacrificed 24-h post injection of dendrimers. Some animals (*n* = 6) were used for the optimization of the dendrimer concentration that was required to infect the cells, in vivo. Another small cohort of animals (*n* = 2) received G4-90/10 by intracranial injection and were sacrificed at 1-week post-injection. One control animal received vehicle by intracranial injections and was sacrificed at 1-week post-injection.

#### 4.3.3. Dendrimer Administration by Intracranial Injection into the Striatum

Mice were anesthetized with isoflurane gas and oxygen and maintained under surgical plane throughout the surgery. The head was shaved and cleaned with chlorhexidine (Molnlycke Healthcare, Gothenburg, Sweden) and appropriately positioned in the ear bar. A midline incision was made on the scalp and the skin was retracted to expose bregma. All mice were injected with 1 µL of G4-90/10, or vehicle at each injection site using a 10 µL Hamilton syringe (Hamilton, Reno, NV, USA) for a total of 4 μL across four different injection sites. Two bilateral burr holes (0.5 mm) were made over the neostriatum at the following coordinates from bregma: +0.5 mm anterior-posterior; ±1.75 mm medial-lateral; and −2.5 mm dorsal-ventral. Injections were carried out at a rate of 0.33 μL/min and the syringe was left in place for three minutes after each injection. After the first injection, the needle was raised by 1 mm and another 1 µL injection was made. The same procedure was then followed on the other hemisphere and the wound was closed using sterile wound clips (7 mm). Mice were placed on a warming bed until fully mobile and were transferred to their recovery cages. The core body temperature and the breathing pattern of the animals were carefully monitored throughout the surgery. The body temperature was maintained at 37 °C, using a heating pad. Post-operative care, including daily monitoring of body weight, was done to the animals for 5 consecutive days.

#### 4.3.4. Dendrimer Administration by Intracarotid Injection through Internal Carotid Artery

Mice were anesthetized with a mixture of isoflurane gas and oxygen. The neck was shaved and cleaned with chlorhexidine (Molnlycke Healthcare) and a small incision was made. Sharp and blunt dissection was used to expose the external carotid artery (ECA), common carotid artery (CCA), and internal carotid artery (ICA). A 6-0 silk suture (MedVet international, Mettawa, IL, USA) was tied around the ECA and just superior of the suture the ECA was cauterized and cut. Then a lose knot was tied around the remainder of the ECA. Microvascular clips were placed on the CCA and the ICA and a slit was made on the ECA between the loose knot and the original suture. A catheter was made using polyethylene tubing with a silica filament inserted at one of its end and joined together using epoxy glue. The polyethylene tubing has an internal diameter of 0.28 mm and external diameter of 0.61 mm. The filament is made of silica (Polymicro technologies, Phoenix, AZ, USA) having beginning internal diameter of 76.9 μm, end internal diameter of 75.5 μm, beginning outer diameter of 154.1 μm, and end outer diameter of 152.1 μm thickness. The filament was then inserted into the slit, the loose knot tightened around the catheter, and the ICA clip was removed. An injection of 4 µL of G4-90/10, or vehicle, was then given through the catheter. After injection, the ICA clip was placed back on and the catheter was removed and the slit in the ECA was sealed by tightening the loose knot and cauterization. Microvascular clips on the ICA and CCA were then removed to allow blood flow to return. The neck was sutured closed using 4-0 silk sutures (MedVet International, Mettawa, IL, USA). Mice were placed on a warming bed until fully mobile, and were transferred to their recovery cages. The core body temperature and the breathing pattern of the animals were carefully monitored throughout the surgery. The body temperature was maintained at 37 °C, using a heating pad. Post-operative care, including daily monitoring of body weight, was done to the animals for 5 consecutive days.

### 4.4. Histology

The animals were sacrificed 24-h and 1-week post-surgery by cervical dislocation based on the group they were assigned to, as mentioned above. Brain, liver, lungs, and spleen were extracted and post-fixed in 4% PFA for 48-h, after which the tissue was transferred to 30% sucrose before being frozen, using 2-methylbutane (Sigma Aldrich, St. Louis, MO, USA). The frozen organs were stored at −80 °C until they were further processed. The tissue was sectioned in cryostat at 30 µm and stained with propidium iodide (PI; 1:1000; Molecular probes, Eugene, OR, USA). The brain tissue, which received the dendrimers through intracranial injection and carotid injection, was sliced in coronal sections and sagittal sections, respectively, to locate the dendrimers in the brain. The coronal sections were used as a positive control to look for the dendrimers in the striatum and the sagittal sections were used to locate the dendrimers in the brain sections, beginning from anterior to posterior ends. To look for dendrimer co-localization with neuronal nuclei and glial cells, rabbit-anti-NeuN antibody (1/500; ab177487 Abcam, Cambridge, UK) and rabbit-anti-GFAP antibody (1/500; ab7260 Abcam, Cambridge, UK) staining was done on different tissue samples collected from the small cohort of animals that received the dendrimers. The sections were mounted on positively charged glass slides, cover-slipped, and viewed on Zeiss Observer inverted microscope, and images were captured using Olympus BX50 Upright Microscope.

## 5. Conclusions

We have shown that a carefully designed dendrimer, with a slight cationic surface/neutral surface (90% OH; 10% NH_2_), is able to cross the BBB when administered through the carotid artery, providing a promising means of effectively delivering therapeutics to the brain.

As mentioned earlier, further investigations into dendrimer migration across the hemispheres through the corpus callosum at different time points should be studied. Secondly, it is important to estimate how long the dendrimers can infect the neurons and glial cells in the brain. Previous studies have used dendrimers to deliver siRNA, miRNA, and other such small RNA molecules. However, use of NH_2_ surface dendrimers are toxic, in vitro and in vivo. Therefore, use of our mixed surface dendrimers offers a safer alternative in terms of cell toxicity and they can be used for various purposes, including delivery of drug or small RNA molecules, in vivo, to the brain via the intra-carotid injection method, which is safer and less invasive than intracranial injections.

## Abbreviations

BBB	Blood–brain barrier
CCA	Common carotid artery
CNS	Central nervous system
DAB	Diaminobutane
DMF	Dimethylformamide
ECA	External carotid artery
EDA	Ethylenediamine
FITC	Fluorescein isothiocyanate
GFAP	Glial fibrillary acidic protein
HBSS	Hank’s balanced salt solution
ICA	Internal carotid artery
IEF	Isoelectric focusing
MEM	minimum essential medium
MWCO	Molecular weight cut-off
MW	Molecular weight
NEAA	Non-essential amine acids
NeuN	Neuronal nuclei
P/S	Penicillin streptomycin
PAMAM	Poly-amidoamine
PAGE	Polyacrylamide gel electrophoresis
PBS	Phosphate buffered saline
PCN	Primary cortical neurons
PFA	Paraformaldehyde
pI	Isoelectric points
PI	Propidium iodide
RP-HPLC	Reverse phase-high performance liquid chromatography
TFA	Trifluoroacetic acid
TLC	Thin layer chromatography
UV	Ultra-violet
